# Dual antiplatelet treatment after coronary artery bypass surgery

**DOI:** 10.1186/1749-8090-10-S1-A102

**Published:** 2015-12-16

**Authors:** Jose Lopez Menendez, Pablo Avanzas, Francisco Callejo, Carlos Morales, Juan C Llosa, Blanca Meana, Jacobo Silva

**Affiliations:** 1 Cardiac Surgery Department, Hospital Universitario Central de Asturias, Oviedo, Spain; 2 Cardiology Department, Hospital Universitario Central de Asturias, Oviedo, Spain

## Background/Introduction

Long-term prognosis after coronary artery bypass grafting (CABG) is related to the patency of coronary grafts, and pathogenesis of graft closure is linked to platelet aggregation. We analyzed the effect on late outcomes of postoperative dual antiplatelet treatment (DAT), maintained during the first year, compared to single antiplatelet treatment (SAT).

## Aims/Objectives

A) Primary: Evaluation of adverse cardiovascular events: Hospital admission for acute coronary syndromes (ACS), unplanned target-vessel revascularization (UTVR), stroke and death of cardiovascular origin. B) Secondary: Evaluation of safety: analysis of bleeding events (BE).

## Method

Retrospective study including all CABG patients during the years 2009-2010, with two years of clinical follow-up. Patients were classified in: A) SAT: daily 100 mg ASA. B) DAT: daily 100 mg ASA plus daily 75 mg clopidogrel.

## Results

The study included 452 patients: 287 SAT (63.5%); 165 DAT (36.5%). 11.9% suffered a primary end-point event; 6.6% ACS; 4.4% UTVR; 1.5% stroke; 3.8% died during follow-up. Safety: 2 (0.4%) suffered a major BE, and 10 (2.2%) minor BE.

DAT was associated with a reduction of the primary end-point from 14.6% to 7.3% (p = 0.020). ACS were reduced from 8.7% to 3.0% (p = 0.020). There were no differences in UTVR nor stroke. Mortality during follow-up was lower in DAT (4.5% versus 2.4%; p = 0.257).

A multivariate Cox proportional-hazards regression was performed; DAT was independently associated with the reduction of events (Hazard ratio 0.49; CI 95% 0.249 - 0.968; p = 0.040).

The greatest benefit of DAT was seen after Off-pump CABG (Hazard ratio 0.395; CI 95% 0.176 - 0.885; p = 0.024) and in diabetic patients (Hazard ratio 0.326; CI 95% 0.124 - 0.854; p = 0.023).

## Discussion/Conclusion

DAT is associated with a reduction of late adverse cardiovascular events after CABG, especially in Off-pump CABG and in diabetic patients. DAT did not increase the risk of BE.

